# Effect of Early Administration of Anti‐MRSA Drugs for Febrile Neutropenia in Allogeneic Hematopoietic Cell Transplantation

**DOI:** 10.1002/jha2.70251

**Published:** 2026-02-17

**Authors:** Anna Akaogi, Junya Kanda, Fumiya Wada, Yasuyuki Arai, Chisaki Mizumoto, Toshio Kitawaki, Kouhei Yamashita, Akifumi Takaori‐Kondo

**Affiliations:** ^1^ Department of Hematology Graduate School of Medicine Kyoto University Kyoto Japan; ^2^ Center for Research and Application of Cellular Therapy Kyoto University Hospital Kyoto Japan

**Keywords:** acute graft‐versus‐host disease, allogeneic‐hematopoietic cell transplantation, anti‐MRSA drugs, febrile neutropenia

## Abstract

**Background:**

In febrile neutropenia (FN), the empirical use of anti‐methicillin‐resistant *Staphylococcus aureus* (MRSA) drugs is recommended, particularly when mucosal damage occurs during fluoroquinolone antibiotic administration. Therefore, the early use of anti‐MRSA drugs may be recommended in many cases of FN after allogeneic hematopoietic cell transplantation (allo‐HCT), but the evidence regarding their efficacy is limited.

**Objectives:**

To assess the impact of early administration of anti‐MRSA drugs on the resolution of fever in patients undergoing allo‐HCT.

**Methods:**

We retrospectively analyzed 186 allo‐HCT patients. Patients receiving anti‐MRSA drugs within 3 days of fever onset formed the early‐decision group; those treated on or after Day 4 or untreated formed the late‐decision group.

**Results:**

The early group showed a significantly shorter fever duration than the late group. (*p* = 0.044). Multivariate logistic regression analysis showed the late group was negatively associated with fever resolution by Day 7 (OR: 0.45, 95% CI: 0.22–0.92, *p* = 0.028). No significant correlation was observed between anti‐MRSA drug timing and acute graft‐versus‐host disease (aGVHD) in the entire cohort. However, among patients aged 51 or older, the late group showed increased risk of aGVHD (HR: 2.22, 95% CI: 1.06–4.64, *p* = 0.034).

**Conclusions:**

Associations were observed between the timing of anti‐MRSA drug administration and clinical outcomes in allo‐HCT, including fever resolution and the incidence of aGVHD in older patients.

**Trial registration:**

The authors have confirmed clinical trial registration is not needed for this submission.

## 1 Introduction

Febrile neutropenia (FN) is a common complication of allogeneic hematopoietic cell transplantation (allo‐HCT), with treatment strategies varying across institutions and cases. The empirical use of anti‐methicillin‐resistant *Staphylococcus aureus* (MRSA) drugs, such as vancomycin, teicoplanin, and daptomycin, is not universally recommended for FN. However, empiric administration is recommended in cases of hemodynamic instability, Gram‐positive cocci detected in blood culture, severe catheter infection or skin and soft tissue infection, prior fluoroquinolone prophylaxis with mucositis, and MRSA or penicillin‐resistant *Streptococcus pneumoniae* infection [1, 2]. Allo‐HCT patients are at high risk for these conditions. Therefore, the early use of anti‐MRSA drugs is recommended in many cases of FN after allo‐HCT. However, in practice, these drugs are often initiated based on blood culture results or as additional therapy when the patient does not respond well to the initial treatment.

In contrast, many cases of Viridans group streptococci (VGS) and *S. aureus* are treated with cefepime, and some authors have questioned the necessity of additional anti‐MRSA drug administration [3]. However, this study was conducted in the general population, and it is not appropriate to apply the results to the patient population of allo‐HCT patients, where delays in appropriate antibiotic administration can be fatal. Against this background, we performed a retrospective analysis to assess the impact of the early administration of anti‐MRSA drugs on the course of FN in allo‐HCT patients. We also analyzed the effects of the timing of anti‐MRSA drug administration on the course of transplantation.

## 2 Methods

### 2.1 Study Cohort and Design

From January 2008 to December 2019, a total of 229 consecutive allo‐HCT patients who did not receive antibiotics on Day 0 and developed FN were included in the study. A total number of 43 patients who recovered from fever within 3 days without receiving anti‐MRSA drugs were excluded. This left 186 patients for the analysis. There were no established criteria for the administration of anti‐MRSA drugs at the facility, and the decision was made by the physician based on individual case evaluations. In principle, Kyoto University Hospital restricts the use of prophylactic antimicrobial agents, including allo‐HCT, for the treatment of hematopoietic malignancies. This restriction is primarily aimed at preventing the emergence of fluoroquinolone‐resistant Gram‐negative bacteria, including multidrug‐resistant *Pseudomonas aeruginosa* [4]. Additionally, evidence that antibiotics do not show a statistically significant difference in all‐cause or infection‐related mortality rates supports this restriction [5]. This study was approved by the Kyoto University Ethics Committee and adhered to the principles of the Declaration of Helsinki. Informed consent was obtained from all the patients. The variables presented in the tables and figures were retrospectively gathered from the patients' medical records.

### 2.2 Endpoints

The primary endpoint was the success rate of fever resolution on Day 7 after the onset of FN. Secondary endpoints included engraftment rate, hospital stay duration, incidence of Grade II–IV acute graft‐versus‐host disease (aGVHD), and non‐relapse mortality (NRM). Additionally, the difference in renal function between the early‐ and late‐decision groups was assessed based on creatinine values on Days 1 and 7 of anti‐MRSA drug administration.

### 2.3 Definitions

Patients receiving anti‐MRSA drugs within 3 days of fever onset formed the early‐decision group. Those treated on or after Day 4 or untreated constituted the late‐decision group. Fever was defined as ≥ 37.5°C, and resolution as maintaining < 37.5°C for 72 h. The analysis only considered the first fever after transplantation. Mucosal disorders include lesions of the oral mucosa and gastrointestinal tract. The severity of lesions was classified according to the CTCAE v5.0 criteria [6], with Grade III or higher disorders being classified as severe and Grade I–II as mild. Patients with lesions in both the oral and gastrointestinal tracts, where either one or both were severe, were classified into the severe group, whereas the others were classified into the mild group.

### 2.4 Statistical Analysis

In terms of patient background, continuous variables were summarized as medians and ranges and compared using the Mann–Whitney *U* test. Categorical variables were summarized as numbers and percentages and compared using Fisher's exact test. Multivariate logistic regression analysis was used to analyze the association between background and fever resolution rate on Day 7 after FN onset. The variables included the timing of anti‐MRSA drug administration, age at transplantation, disease type (myeloid vs. lymphoid), transplant source, conditioning intensity, disease stage, and HLA compatibility (full match vs. others). GVHD prophylaxis and ATG use were not included as covariates because cyclosporine and ATG were used in only a small number of cases (8.1% and 2.7% of the total cohort, respectively). The incidences of fever resolution, aGVHD, engraftment rate, discharge, and NRM were analyzed using cumulative incidence methods; death without event was defined as a competing factor for the incidences of fever resolution, aGVHD, engraftment rate, and discharge, and relapse was defined as a competing factor for NRM. These were compared using Gray's test. Fine and Gray proportional hazards models were used for multivariate analysis. For the analysis of FN, follow‐up for each patient was defined as the time from FN onset to confirmed fever resolution, or to the date of death if fever resolution was not achieved. Serum creatinine levels on Day 1 and Day 7 of anti‐MRSA drug administration were compared between the early and late‐decision groups using the Mann–Whitney *U* test. Statistical significance was set at *p* < 0.05. R (version 3.1.1; R Development Core Team) and EZR (Saitama Medical Center, Jichi Medical University, Saitama, Japan) were used for statistical analyses [7].

## 3 Result

### 3.1 Patient Characteristics

Table 1 presents the clinical characteristics of the entire patient cohort and compares the early and late anti‐MRSA drug‐decision groups. The early‐decision group consisted of 91 patients, and the late‐decision group consisted of 95 patients. The underlying diseases included acute myeloid leukemia (74 patients), acute lymphoblastic leukemia (30 patients), chronic myeloid leukemia (7 patients), myelodysplastic syndrome (27 patients), and other malignancies. The median age at HCT was 55 years (range: 17–70), with 112 males and 74 females. The disease status was complete remission (CR) in 118 patients, non‐CR in 36, and no high‐intensity chemotherapy treatment in 32. The conditioning regimens included myeloablative conditioning (MAC) in 109 patients and reduced‐intensity conditioning (RIC) in 77. The transplant sources were the bone marrow (101 cases), peripheral blood (18 cases), and cord blood (67 cases), with HLA matching in 95 cases and mismatch in 91 cases. Acute GVHD prophylaxis included cyclosporine in 15, tacrolimus in 171, and ATG in 5 patients. Age and sex varied between the early‐ and late‐decision groups (*p *< 0.001 and 0.036), but the other characteristics were similar.

**TABLE 1:** Patient characteristics.

Characteristics			Overall (*n* = 186)	Early decision group (*n* = 91)	Late decision group (*n* = 95)	*p* value
Age at transplant			55.00 [17.00, 70.00]	44.00 [17.00, 68.00]	55.00 [20.00, 70.00]	< 0.001^***^
Recipient sex	Female		74 (39.8)	29 (31.9)	45 (47.4)	0.036^*^
	Male		112 (60.2)	62 (68.1)	50 (52.6)	
Disease	AML		74 (39.8)	40 (44.0)	34 (35.8)	0.657
	ALL		30 (16.1)	14 (15.4)	16 (16.8)	
	CML		7 (3.8)	4 (4.4)	3 (3.2)	
	MDS		27 (14.5)	10 (11.0)	17 (17.9)	
	MPAL		1 (0.5)	1 (1.1)	0 (0.0)	
	NHL		24 (12.9)	10 (11.0)	14 (14.7)	
	HL		2 (1.1)	1 (1.1)	1 (1.1)	
	ATL		11 (5.9)	5 (5.5)	6 (6.3)	
	Other lymphoma		4 (2.2)	1 (1.1)	3 (3.2)	
	Plasma cell neoplasm		2 (1.1)	2 (2.2)	0 (0.0)	
	Other		4 (2.2)	3 (3.3)	1 (1.1)	
Source of stem cells	Bone marrow		101 (54.3)	49 (53.8)	52 (54.7)	0.542
	Peripheral blood		18 (9.7)	11 (12.1)	7 (7.4)	
	Cord blood		67 (36.0)	31 (34.1)	36 (37.9)	
Conditioning regimen	Myeloablative		109 (58.6)	59 (64.8)	50 (52.6)	0.103
		CY + TBI regimen	79 (42.5)	47 (51.6)	32 (33.7)	
		Other TBI regimen	1 (0.5)	0 (0.0)	1 (1.1)	
		BU + CY regimen	11 (5.9)	6 (6.6)	5 (5.3)	
		Other non TBI regimen	18 (9.7)	6 (6.6)	12 (12.6)	
	Reduced intensity		77 (41.4)	32 (35.2)	45 (47.4)	
		FL + BU regimen	7 (9.1)	5 (15.6)	2 (4.4)	
		FL + CY regimen	1 (1.3)	0 (0.0)	1 (2.2)	
		FL + Mel regimen	68 (88.3)	27 (84.4)	41 (91.1)	
		Other regimen	1 (1.3)	0 (0.0)	1 (2.2)	
GVHD prophylaxis		CyA based	15 (8.1)	8 (8.8)	7 (7.4)	0.792
		Tac based	171 (91.9)	83 (91.2)	88 (92.6)	
Use of ATG		No	181 (97.3)	90 (98.9)	91 (95.8)	0.369
		Yes	5 (2.7)	1 (1.1)	4 (4.2)	
Disease status		CR	118 (63.4)	61 (67.0)	57 (60.0)	0.63
		Non CR	36 (19.4)	16 (17.6)	20 (21.1)	
		Untreated	32 (17.2)	14 (15.4)	18 (18.9)	
HLA compatibility		Match	95 (51.1)	47 (51.6)	48 (50.5)	0.885
		Mismatch	91 (48.9)	44 (48.4)	47 (49.5)	

Abbreviations: AML, acute myeloid leukemia; ALL, acute lymphoblastic leukemia; ATL, adult T‐cell leukemia‐lymphoma; BU, busulfan; CML, chronic myeloid leukemia; CR, complete remission; CY, cyclophosphamide; CyA, cyclosporine; FL, fludarabine; GVHD, graft versus host‐disease; HL, Hodgkin's lymphoma; HLA, human leukocyte antigen; MDS, myelodysplastic syndrome; Mel, melphalan; MPAL, mixed phenotype acute leukemia; NHL, non‐Hodgkin's lymphoma; Tac, tacrolimus; TBI, total body irradiation.

* *p *< 0.05

*** *p *< 0.001

### 3.2 Success Rate of Fever Resolution in all Patients

**FIGURE 1:**
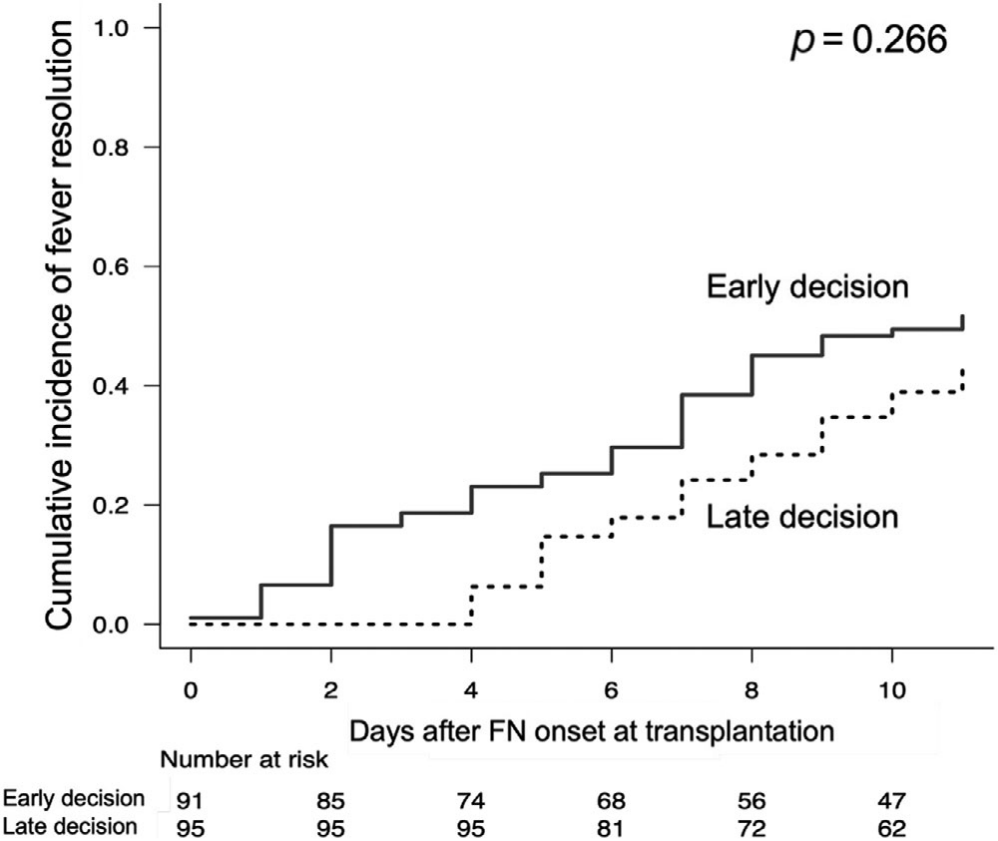
Effect of early or late decision of anti‐MRSA drug administration on fever resolution.

The median number of days from hematopoietic cell infusion to FN onset was 5 days (range: 1–19 days) in the early‐decision group and 6 days (range: 1–27 days) in the late‐decision group, with no significant difference between the two groups (*p* = 0.23). The number of days from FN onset to fever resolution was significantly shorter in the early‐decision group than in the late‐decision group (median 11.0 vs. 12.0 days, *p* = 0.044) (Figure S1). The cumulative incidence of fever resolution on Day 7 after FN onset in the entire cohort was 38.5% (95% CI: 29%–48%) in the early‐decision group and 24.2% (95% CI: 16%–33%) in the late‐decision group (Figure 1), with no significant difference (*p *= 0.266). In the multivariate analysis, a negative correlation was found between the late‐decision group and successful resolution (OR: 0.45 [95% CI 0.22–0.92], *p* = 0.028) (Table 2).

**TABLE 2:** Clinical features affecting fever subsides in univariate and multivariate analysis.

Variable		Univariate		Multivariate	
		OR (95% CI)	*p* value	OR (95% CI)	*p* value
Anti‐MRSA drug administration	Early decision	1	Reference	1	Reference
	Late decision	0.51 (0.27−0.96)	0.037^*^	0.45 (0.22−0.92)	0.028^*^
Age at transplant (continuous)		1.02 (1.00−1.04)	0.099	1.03 (1.00−1.06)	0.044^*^
Disease status	CR	1	Reference	1	Reference
	Non CR	0.47 (0.19−1.14)	0.096	0.49 (0.19−1.29)	0.149
	Untreated	1.94 (0.89−4.25)	0.095	1.47 (0.59−3.66)	0.403
Source of stem cell	Bone marrow	1	Reference	1	Reference
	Peripheral blood	3.12 (1.16−8.39)	0.024^*^	3.30 (1.08−10.10)	0.037^*^
	Cord blood	0.51 (0.26−1.01)	0.054	1.12 (0.39−3.23)	0.835
Disease	Myeloid	1	Reference	—	—
	Lymphoid	0.60 (0.31−1.15)	0.124	—	—
Conditioning	MAC	1	Reference	—	—
	RIC	1.50 (0.80−2.81)	0.201	—	—
HLA	Match	1	Reference	1	Reference
	Mismatch	0.52 (0.28−0.99)	0.045^*^	0.51 (0.20−1.31)	0.164
Mucosal disorder	Mild	1	Reference	1	Reference
	Severe	1.92 (1.01−3.62)	0.045^*^	1.91 (0.94−3.90)	0.076

Abbreviations: CR, complete remission; HLA, human leukocyte antigen; MAC, myeloablative conditioning; RIC, reduced intensity conditioning.

* *p *< 0.05

### 3.3 Success Rate of Fever Resolution Stratified by Patient and Transplant Backgrounds

Multivariate analyses were performed according to the degree of mucosal damage, age, disease status, HLA compatibility, and source of transplantation (Table 3). The results showed lower resolution rates in the late‐decision group, regardless of patient background, except in cases of cord blood transplantation; however, most of these differences were not statistically significant. In cases with severe mucosal disorders, the odds ratio in the late‐decision group was lower than that in the analysis of the entire cohort (OR: 0.36 [0.12–1.11], *p* = 0.075).

**TABLE 3:** Analysis of the effect of early or late decision of anti‐MRSA drugs administration on fever resolution by stratification of patient background.

Subgroup		No. of events	OR (95% CI)	*p* value
Mucosal disorder	Mild	119	0.59 (0.22−1.56)	0.288
	Severe	67	0.36 (0.12−1.11)	0.075
Age at transplant	< 51	93	0.46 (0.15−1.37)	0.162
	≥ 51	93	0.44 (0.17−1.16)	0.097
Disease Status	CR	118	0.42 (0.17−1.00)	0.049^*^
	Non CR	36	—	—
	Untreated	32	0.72 (0.12−4.48)	0.723
Source of stem cells	Bone marrow	101	0.28 (0.11−0.75)	0.011^*^
	Peripheral blood	18	0.21 (0.01−3.26)	0.263
	Cord blood	67	1.12 (0.29−4.29)	0.87
HLA compatibility	Match	95	0.31 (0.11−0.81)	0.018^*^
	Mismatch	91	0.68 (0.23−1.97)	0.474

Abbreviations: CR, complete remission; HLA, human leukocyte antigen.

* *p *< 0.05

### 3.4 Days to Engraft and Discharge

For engraftment and discharge after FN onset, the cumulative incidence rates of the late‐decision group were compared with those of the early‐decision group. On the Day 30 after the onset of fever, the cumulative incidence rates of engraftment were 87.8% in the early‐decision group (95% CI: 79%–93%) and 86.3% in the late‐decision group (95% CI: 78%–92%), with no significant difference (*p* = 0.332). At the Day 60, the cumulative discharge rates were 38.5% in the early‐decision group (95% CI: 29%–48%) and 43.2% in the late‐decision group (95% CI: 33%–53%); however, the difference was not significant (*p* = 0.272).

### 3.5 Acute GVHD Incidence

On Day 100 from FN onset, the cumulative incidence of Grade II–IV aGVHD was 38.8% (95% CI: 29%–49%) in the early‐decision group and 47.4% (95% CI: 37%–57%) in the late‐decision group (*p* = 0.123) (Figure S2). Multivariate analysis showed an HR of 1.42 (95% CI: 0.92–2.19, *p* = 0.12) for aGVHD in the late‐decision group compared to the early‐decision group (Table 4). No significant correlation was observed between the timing of MRSA drug administration and the development of aGVHD in the entire cohort. However, in a stratified analysis, the incidence of Grade II–IV aGVHD was significantly higher in the late‐decision group in patients aged 51 years or older (HR: 2.22 [1.06–4.64], *p* = 0.034) (Table 5).

**TABLE 4:** Univariate and multivariate analysis of factors associated with grades II–IV aGVHD.

Variable		Univariate		Multivariate	
		HR (95% CI)	*p* value	HR (95% CI)	*p* value
Anti‐MRSA drug administration	Early decision	1	Reference	1	Reference
	Late decision	1.39 (0.90−2.15)	0.14	1.42 (0.92−2.19)	0.12
Age at transplant (continuous)		1.00 (0.99−1.02)	0.76	—	—
Disease status	CR	1	Reference	—	—
	Non CR	0.94 (0.54−1.63)	0.836	—	—
	Untreated	1.18 (0.70−1.99)	0.54	—	—
Source of stem cell	Bone marrow	1	Reference	—	—
	Peripheral blood	0.53 (0.22−1.29)	0.16	—	—
	Cord blood	1.33 (0.86−2.04)	0.2	—	—
HLA compatibility	Match	1	Reference	1	Reference
	Mismatch	1.58 (1.02−2.44)	0.041^*^	1.6 (1.03−2.49)	0.036^*^
Conditioning regimen	MAC	1	Reference	—	—
	RIC	1.06 (0.68−1.65)	0.79	—	—
Disease	Myeloid	1	Reference	—	—
	Lymphoid	1.15 (0.74−1.78)	0.53	—	—
Mucosal disorder	Mild	1	Reference	—	—
	Severe	0.71 (0.44−1.14)	0.15	—	—

Abbreviations: CR, complete remission; HLA, human leukocyte antigen; MAC, myeloablative conditioning; RIC, reduced intensity conditioning.

* *p *< 0.05

**TABLE 5:** Analysis of the effect of early or late decision of anti‐MRSA drugs administration on grade II–IV aGVHD by stratification of patient background.

Subgroup		No. of events	HR (95% CI)	*p* value
Mucosal disorder	Mild	119	1.43 (0.84−2.44)	0.19
	Severe	67	1.25 (0.56−2.76)	0.58
Age	< 51	93	0.86 (0.44−1.69)	0.67
	≥ 51	93	2.22 (1.06−4.64)	0.034^*^
Disease Status	CR	118	1.06 (0.61−1.84)	0.84
	Non CR	36	1.70 (0.55−5.24)	0.36
	Untreated	32	2.74 (0.84−8.96)	0.1
Source of stem cell	Bone marrow	101	1.81 (0.96−3.40)	0.066
	Peripheral blood	18	1.04 (0.19−5.82)	0.96
	Cord blood	67	1.10 (0.54−2.05)	0.89
HLA compatibility	Match	95	1.25 (0.64−2.43)	0.52
	Mismatch	91	1.53 (0.86−2.70)	0.15

Abbreviations: CR, complete remission; HLA, human leukocyte antigen.

* *p *< 0.05

### 3.6 Blood Culture Results

In Tables S1 and S2, the blood culture outcomes of the initial FN episodes are described for the 186 patients included in the study. Among them, 58 had positive blood cultures. In the early anti‐MRSA medication group, 33 of 91 patients had positive blood cultures, whereas in the late‐decision group, 25 of 95 patients had positive cultures. The positivity rate did not differ significantly between the groups (*p* = 0.157). The total number of positive cultures was 79, with 46 Gram‐positive, 32 Gram‐negative, and one fungus (*Candida albicans*). Gram‐positive bacteria were significantly more prevalent in the early‐decision‐making group (*p* < 0.001). The reasons for deciding to administer anti‐MRSA drugs in the early‐decision group are shown in Table S3. In addition to blood culture results, these reasons included the presence of mucosal damage, focal signs of infection, such as in the skin or central venous catheter, hemodynamic instability, the physician's judgment that fever resolution was delayed, and a history of MRSA or Vancomycin‐resistant *Enterococcus* infection.

The study also assessed fever resolution rates while excluding cases in which decisions on anti‐MRSA drug administration based on blood culture results were made within 3 days of fever onset. Thus, in this cohort, the decision regarding the initiation and timing of anti‐MRSA drugs was made based on factors other than blood culture results. This analysis covered 149 cases, removing 19 from the early‐decision group, in which Gram‐positive bacteria led to anti‐MRSA administration, and 18 from the late‐decision group, in which Gram‐negative bacteria led to withholding. The results showed that the rate of fever resolution tended to be lower in the late‐decision group (OR: 0.45 [0.21–1.01], *p* = 0.052) (Table S4). In the stratified analysis, fever resolution rates were lower in the late‐decision group, regardless of patient background (Table S5). Similarly, when examining the impact of anti‐MRSA drug timing on the development of Grade II–IV aGVHD in this cohort, no association was observed in all patients (Table S6). However, patients aged 51 years or older and those with HLA mismatch in the late anti‐MRSA decision group showed a significant increase in aGVHD incidence (HR: 2.66, 95% CI: 1.23–5.77, *p* = 0.013, and HR: 1.85, 95% CI: 1.06–3.23, *p* = 0.031, respectively.) (Table S7).

### 3.7 Empiric Antibiotic Therapy

In the analyzed cohort, the *β*‐lactam antibiotics used included cefepime, meropenem, and piperacillin/tazobactam. There was no significant difference between the early‐ and late‐decision groups in the use of *β*‐lactam antibiotics with anaerobic coverage (meropenem and piperacillin/tazobactam) (*p* = 0.814).

### 3.8 NRM

The incidence of NRM at Day 100 after FN onset was 8.9% (95% CI: 4.1%–15.7%) in the early‐decision group and 5.3% (95% CI: 2.0%–11.2%) in the late‐decision group (*p* = 0.329). No significant differences were observed between the two groups (Figure S3).

### 3.9 Renal Function

The median serum creatinine level was 0.63 and 0.60 mg/dL in the early‐decision group and late‐decision group, respectively, on Day 1 of anti‐MRSA drug administration, with no significant difference between the two groups (*p* = 0.327). On Day 7, the median serum levels were 0.63 and 0.59 mg/dL in the early and late groups, respectively. Similarly, there was no significant difference between the two groups (*p* = 0.49) (Figure S4).

## 4 Discussion

In this study, we demonstrated that an early decision to administer anti‐MRSA drugs results in the rapid resolution of fever in FN after allo‐HCT. We consider early fever resolution to be an important indicator reflecting the early suppression of inflammation in FN. Furthermore, it should be regarded as a clinically meaningful outcome in terms of ensuring careful systemic management after transplantation and relieving patient discomfort at an early stage. Stratified analysis revealed higher resolution rates in the early‐decision group, regardless of patient background, except in cases involving cord blood transplantation. Additionally, among patients aged 51 years or older, the incidence of Grade II–IV acute GVHD was significantly lower in the early‐decision group, suggesting that older individuals may benefit from early administration of anti‐MRSA drugs.

A controlled study comparing persistent FN patients who were administered either placebo or placebo with added anti‐MRSA drugs showed no difference in the fever resolution rate [8, 9]. Similarly, the Japanese guidelines state that even in cases where fever persists 3 to 4 days after the onset of FN, the same initial antibiotics can be continued if there are no other significant findings besides fever and the patient is in good condition [10]. In contrast, in our study, the group that initiated anti‐MRSA medication within 3 days of fever onset had a significantly higher rate of fever resolution on FN Day 7 than the group that received it after 4 days of fever or did not receive it. This disparity in results may be attributed to the differences in patient populations, as our study included only allo‐HCT cases. The median time to fever resolution in our study cohort was 12 days, which differed from the median time to fever resolution of 5 days in the general FN population [11]. Patients receiving fluoroquinolone prophylaxis were excluded from this study because of their small sample size. These results suggest that early administration of anti‐MRSA drugs may be effective in allo‐HCT even in the absence of fluoroquinolone administration. Because infections treated with fluoroquinolone prophylaxis are more likely to be caused by Gram‐positive bacteria [12], an earlier decision to administer anti‐MRSA drugs is likely to contribute to a more frequent resolution of fever in centers where fluoroquinolone prophylaxis is administered.

Several factors contribute to early fever resolution in allo‐HCT‐FN with the early administration of anti‐MRSA drugs. Previous reports indicate that the breakdown of blood cultures in early allo‐HCT is 50.6%–52.5% Gram‐positive and 41.6%–47.5% Gram‐negative bacteria [13, 14], highlighting the importance of vigilance for Gram‐positive bacteria. Additionally, the insertion of central venous catheters in almost all patients increases the risk of Gram‐positive bacterial infections, including *S. aureus* and *Staphylococcus epidermidis*. Furthermore, in allo‐HCT, despite the small number of cases that do not experience fever at the time of engraftment, bacteremia occurs in only 25%–53.3% of cases [13, 14, 15, 16]. Even in our study cohort, in which all patients developed FN, 31.0% of patients had positive blood cultures at the time of engraftment, which is consistent with previous reports. Thus, in more than half of the cases, the organisms were not identified by blood culture, and the decision to add antibiotics based solely on blood culture results was not an appropriate strategy. In the early‐decision group, the detection rate of Gram‐positive bacteria was significantly higher than in the late‐decision group. This high detection rate suggests that, in many cases, the prediction of Gram‐positive bacterial infections based on clinical findings, including mucosal damage, was correct. To eliminate the influence of biases from blood culture results, we conducted an analysis excluding cases where the decision to administer anti‐MRSA drugs was based on blood culture results, focusing on 149 cases. The results showed that, even in this group, early administration of anti‐MRSA drugs contributed to early fever resolution. Therefore, it is worthwhile to administer anti‐MRSA drugs without waiting for the blood culture results.

The fact that patients with severe mucosal disorders benefit from early administration of anti‐MRSA drugs may be related to infections caused by commensal bacteria in the oral and gastrointestinal tracts. Barrier failure due to damage to the oral mucosa can act as a gateway for VGS, a commensal organism in the oral cavity. In the gastrointestinal tract, problems caused by mucosal barrier failure are related to enteric bacteria in the gastrointestinal tract, including *Enterococcus* spp. VGS and *Enterococcus faecium*, among enterococci, are important causes of severe infections in neutropenic hosts [17, 18]. The presence of penicillin‐resistant strains of VGS and *E. faecium* may increase the need for anti‐MRSA drug therapy in allo‐HCT cases with barrier failure.

Furthermore, the lower incidence of Grade II–IV aGVHD in the early‐decision group of patients aged 51 years or older may be due to the early decline of FN‐induced cytokines, such as IL‐2R*α*, TNFR‐1, HGF, and IL‐8, which are known biomarkers of aGVHD. In particular, high serum IFN*γ* levels on Day 6 of transplantation are considered to be a risk factor for the development of aGVHD [19]. Early resolution of fever and suppression of inflammatory cytokines may decrease the incidence of aGVHD.

This study has several limitations. First, one limitation of this study is the potential for lead time bias: by definition, patients in the late‐decision group received anti‐MRSA drugs later, making a direct comparison of the number of days from fever onset to resolution between the early‐ and late‐decision groups inherently unfair. To support our findings, we conducted an additional analysis using a Cox proportional hazards model in which anti‐MRSA drug administration was treated as a time‐dependent covariate. As a result, the fever resolution on FN Days 8, 9, 10, or 11 was significantly associated with the use of anti‐MRSA drugs (data not shown). These findings support the notion that anti‐MRSA therapy is effective in patients undergoing allo‐HCT and that many of these patients may have a latent need for such treatment. Second, this was a single‐center retrospective study and may have included an insufficient number of cases. Finally, the efficacy of the antibiotic treatment was assessed on Day 7 after the onset of fever; however, there may be room for debate regarding the validity of this timing for assessing treatment effects. However, a sensitivity analysis was conducted, and consistent results were obtained when investigating the success rate of fever resolution at Days 8, 9, 10, and 11 after fever onset (data not shown). Analysis of the fluoroquinolone prophylaxis group, which may benefit from earlier administration of anti‐MRSA drugs owing to a higher incidence of Gram‐positive bacteria, is not yet available and requires further study.

In conclusion, early administration of anti‐MRSA agents was associated with a shorter duration of fever in patients with FN following allo‐HCT. Moreover, early administration of anti‐MRSA drugs may be associated with a lower incidence of Grade II–IV aGVHD in older patients, suggesting that the timing of anti‐MRSA therapy could be clinically relevant in this population. Early administration of anti‐MRSA drugs may be relevant not only to the course of FN but also to the overall post‐transplant course, including the development of aGVHD.

## Author Contributions

Conceptualization and design of the study: Anna Akaogi and Junya Kanda. All the authors have made substantial contributions to the design of the work, the analysis and interpretation of the data, and the drafting or revision of the manuscript. All authors have approved the final manuscript for publication.

## Funding

This work was supported in part by the JSPS KAKENHI Grant Number 24K11515 (JK) and the Takeda Science Foundation (JK).

## Ethics Statement

This study was approved by the Kyoto University Ethics Committee and adhered to the principles of the Declaration of Helsinki.

## Consent

Informed consent was obtained from all the patients.

## Conflicts of Interest

The authors declare no conflicts of interest.

## Supporting information




**Supporting Figure 1**: jha270251‐sup‐0001‐figuresS1‐S4.pdf

**Supporting Table 1**: jha270251‐sup‐0001‐tablesS1‐S7.xlsx

## Data Availability

The datasets used in this study may contain personal information and are therefore not publicly available. Data may be provided by the corresponding author upon reasonable request.
