# Supplementary material for: Effect of Early Administration of Anti‐MRSA Drugs for Febrile Neutropenia in Allogeneic Hematopoietic Cell Transplantation
Source: EJHaem. 2026 Feb 17;7(1):e70251. doi: 10.1002/jha2.70251 (PMC12910238; doi:10.1002/jha2.70251)
Supplement: Supplementary file 1 — Supporting Figure 1: jha270251‐sup‐0001‐figuresS1‐S4.pdf [file JHA2-7-e70251-s001.pdf]

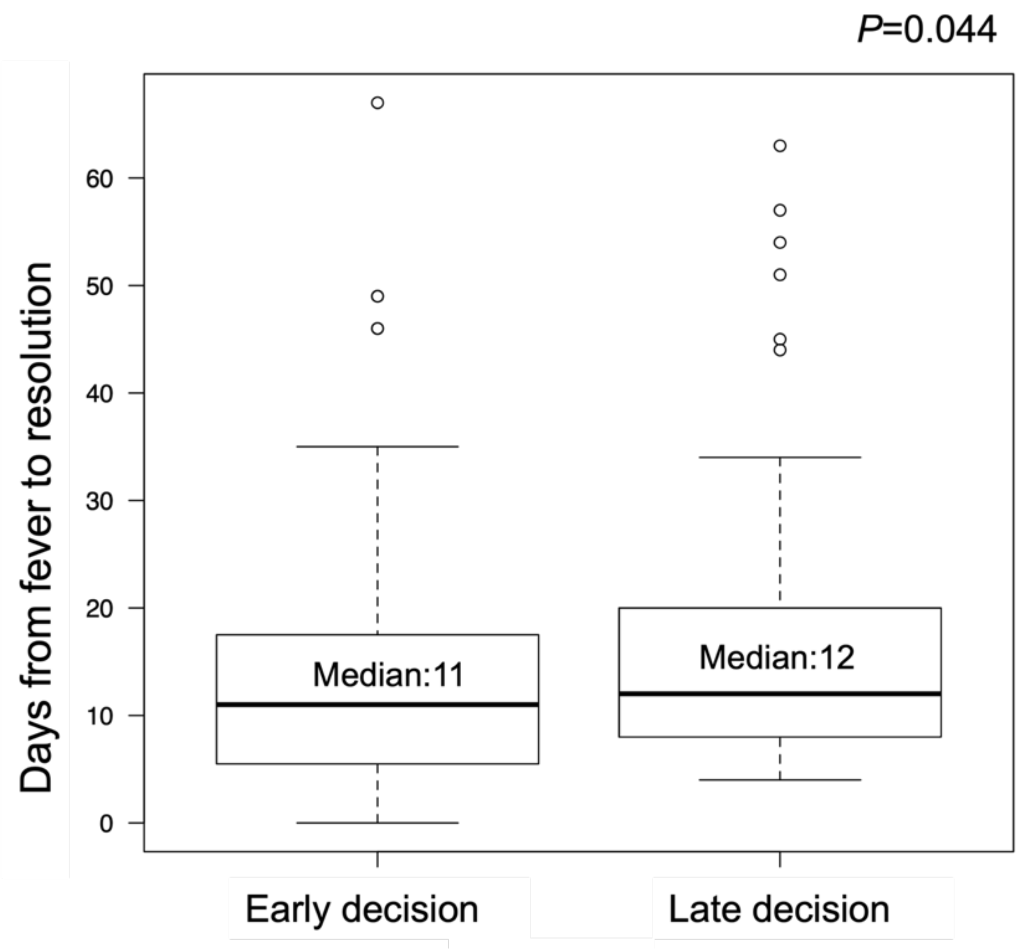

Supplemental Figure 1. Days from fever to resolution on early or late decision of anti-MRSA drug administration.

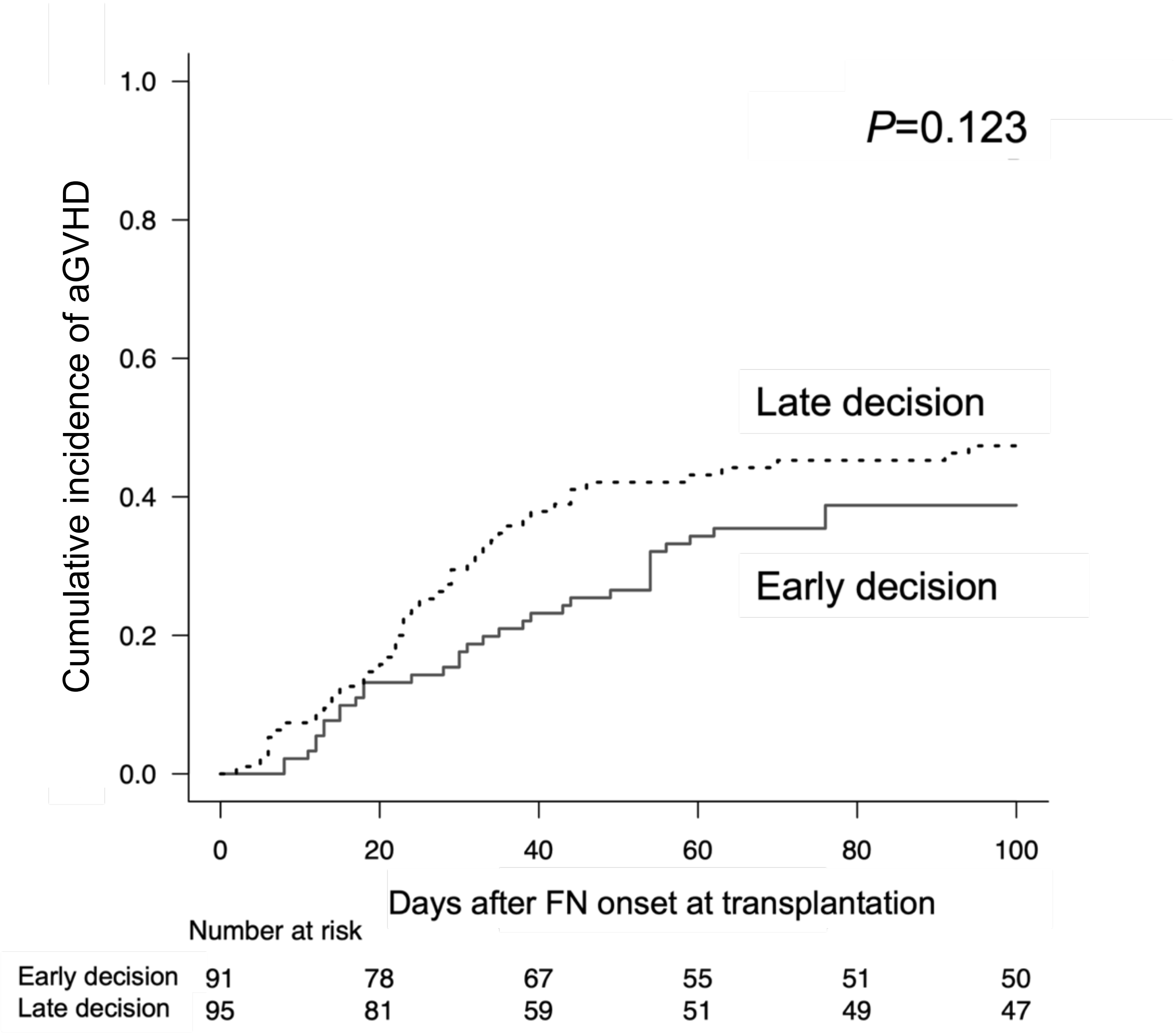

Supplemental Figure 2. Effect of early or late decision of anti-MRSA drug administration on aGVHD.

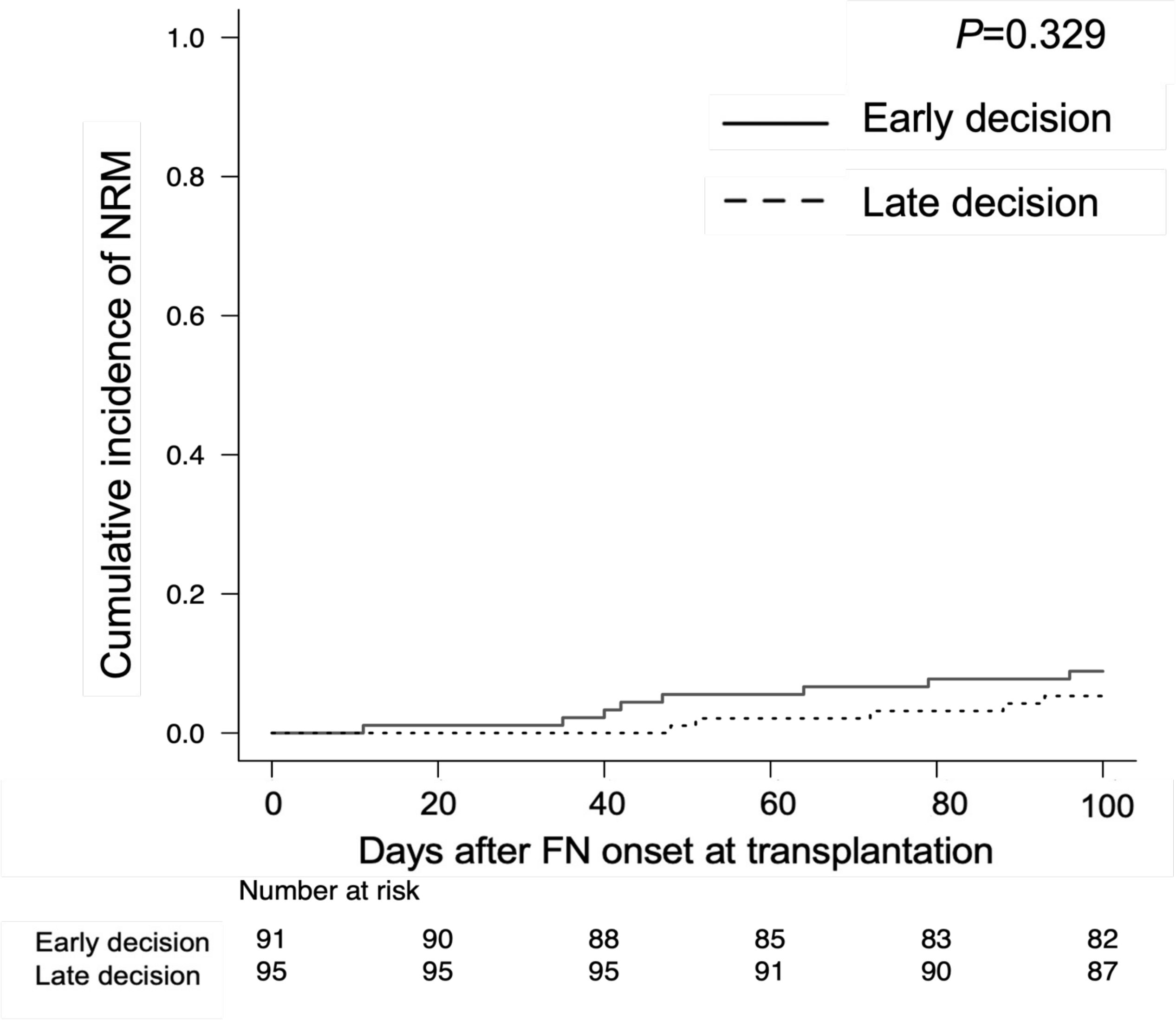

Supplemental Figure 3. Comparison of NRM between the early and late decision groups.

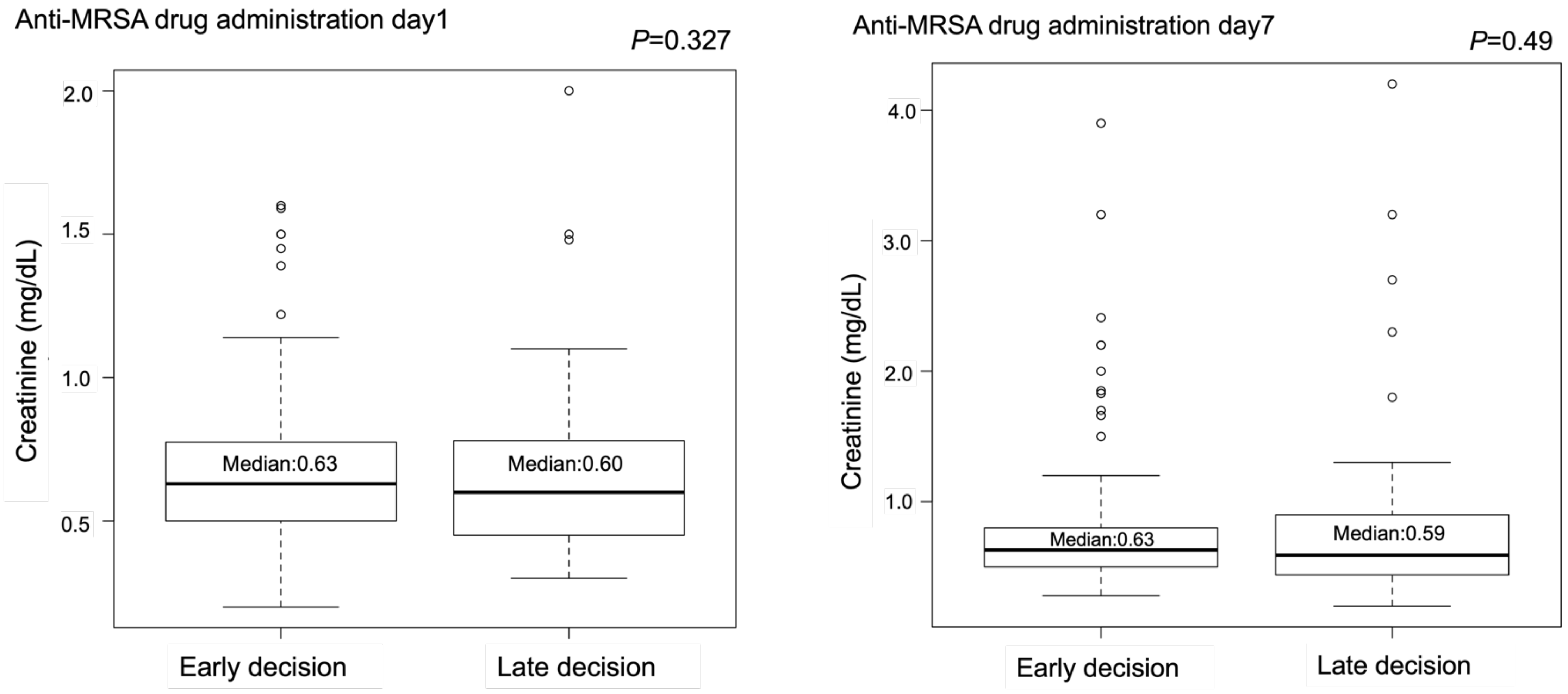

Supplemental Figure 4. Comparison of renal function between early and late decision groups on day 1 and day 7 of anti-MRSA drug administration.
